# Vaccination of sows against type 2 Porcine Reproductive and Respiratory Syndrome Virus (PRRSV) before artificial insemination protects against type 2 PRRSV challenge but does not protect against type 1 PRRSV challenge in late gestation

**DOI:** 10.1186/1297-9716-45-12

**Published:** 2014-02-02

**Authors:** Kiwon Han, Hwi Won Seo, Changhoon Park, Chanhee Chae

**Affiliations:** 1 Department of Veterinary Pathology, College of Veterinary Medicine, Seoul National University, 1 Gwanak-ro, Gwanak-gu, Seoul 151-742, Republic of Korea

## Abstract

The objective of the present study was to determine the effects of the commercially available type 2 Porcine Reproductive and Respiratory Syndrome Virus (PRRSV)-based modified live vaccine against type 1 and type 2 PRRSV challenge in pregnant sows. Half of the sows in the study were vaccinated with a type 2 PRRSV-based vaccine 4 weeks prior to artificial insemination while the other half remained non-vaccinated. Sows were then challenged intranasally with type 1 or type 2 PRRSV at 93 days of gestation. The sows which received the type 2 PRRSV-based vaccine followed by type 2 PRRSV challenge had significantly higher neutralizing antibody titers against type 2 PRRSV than they did against type 1 PRRSV. These same sows had higher frequencies of IFN-γ-secreting cells when stimulated with type 2 PRRSV compared to those stimulated with type 1 PRRSV. Subsequent virological evaluation demonstrated that the type 2 PRRSV-based vaccine reduced the type 2 PRRSV load but not the type 1 PRRSV load present in the blood of the sows. Additionally, vaccination of pregnant sows with the type 2 PRRSV-based vaccine effectively reduced the level of type 2 PRRSV nucleic acids observed in fetal tissues from type 2 PRRSV-challenged sows but did not reduce the level of type 1 PRRSV nucleic acid observed in fetal tissues from type 1 PRRSV-challenged sows. This study demonstrates that the vaccination of pregnant sows with the type 2 PRRSV-based vaccine protects against type 2 PRRSV challenge but does not protect against type 1 PRRSV challenge.

## Introduction

Porcine Reproductive and Respiratory Syndrome (PRRS) Virus (PRRSV) is a widely disseminated and economically important swine virus that is known to cause reproductive failure in pregnant sows and respiratory disease in nursery and grower/finishing pigs [1]. In the early 1990s, all European PRRSV isolates were closely related and all North American isolates were also closely related, however, the two groups were distant from one another [2-4]. Later, genetic analysis defined the two main genotypes of PRRSV: type 1 (European-like) and type 2 (North American-like) [3,5]. Type 1 and type 2 PRRSV differ significantly in terms of their clinical, genetic, and antigenic aspects [6-8]. At the present time, type 1 PRRSV is also found in both North American and Asian countries [9-12].

The commercial modified live virus (MLV) vaccine (Ingelvac® PRRS MLV, Boehringer Ingelheim Vetmedica Inc., St. Joseph, MO, USA) based on type 2 PRRSV was first licensed for worldwide use in 3 to 18-week-old pigs in 1994 and in pregnant female breeding-stock pigs in 1996. This MLV vaccine has been used extensively by swine producers to protect pigs against PRRSV infection across the globe. Cross-protection conferred by the type 2 PRRSV-based vaccine against type 1 PRRSV is a major issue because of the co-existence of type 1 and type 2 PRRSV in many Asian countries [10-12]. However, no peer-reviewed studies have assessed the efficacy of the type 2 PRRSV-based vaccine against type 1 and type 2 PRRSV in pregnant gilts. Therefore, the objective of this study was to determine the effects of the type 2 PRRSV-based vaccine against type 1 and type 2 PRRSV challenge in pregnant sows, using clinical, immunological, virological, and pathological measures for evaluation.

## Materials and methods

### PRRSV inocula

Type 1 (SNUVR090485) and type 2 (SNUVR100059) PRRSV were used as inocula. The SNUVR090485 virus was isolated from lung samples from an aborted fetus in 2009 in the Kyounggi Province. The SNUVR100059 was isolated from lymph node samples of an aborted fetus in 2009 in the Chungcheung Province. The nucleotide sequence homology in open reading frame (ORF) 5 between the type 1 PRRSV (SNUVR090485, Genbank no. JN315686) and the vaccine strain (Genbank no. AF535152) is 68% and between the type 2 PRRSV (SNUVR100059, Genbank no. JX988620) and the vaccine strain is 84%. Sequence homology was determined using BioEdit version 7.0.0 (Ibis Biosciences, Carlsbad, CA, USA) [13].

### Experimental design

Twenty-six seronegative sows (parity = 2) were purchased from a PRRSV-free herd. All sows were moved to a research facility, housed individually in separate rooms, and randomly allocated into 6 groups: the vaccinated and type 1 PRRSV-challenged group (group 1, *n* = 5), the vaccinated and type 2 PRRSV-challenged group (group 2, *n* = 5), the non-vaccinated and type 1 PRRSV-challenged group (group 3, *n* = 5), the non-vaccinated and type 2 PRRSV-challenged group (group 4, *n* = 5), the vaccinated and non-challenged group (group 5, *n* = 3), and the negative control group which was non-vaccinated and non-challenged (group 6, *n* = 3) (Table 1).

**Table 1:** Study design with vaccination and challenge statuses of PRRSV

**Group ( *n *)**^**a**^	**Vaccination**	**Challenge**	
		**Type 1 PRRSV**	**Type 2 PRRSV**
1 (5)	O	O	X
2 (5)	O	X	O
3 (5)	X	O	X
4 (5)	X	X	O
5 (3)	O	X	X
6 (3)	X	X	X

^a^*n*, number of sows in group.

The estrous cycles of all sows were synchronized as previously described [14]. Sows in groups 1, 2, and 5 were intramuscularly vaccinated with a 2.0 mL dose of the type 2-based PRRSV vaccine (Ingelvac® PRRS MLV, Boehringer Ingelheim Vetmedica Inc.) 4 weeks prior to artificial insemination, according to the manufacturer's instructions**.** Sows from all 6 groups were inseminated with 80 mL of extended semen every 24 h for 3 days. The sows were then monitored for signs of estrus, and any sows that recycled were re-inseminated at 24 h intervals for 3 days. At approximately 5 and 8 weeks post artificial insemination, pregnancy was confirmed with ultrasonography. Sows were then allowed to gestate and carry the pregnancy to term.

At 3 weeks antepartum (93 days of gestation), the vaccinated sows from group 1 and the non-vaccinated sows from group 3 were inoculated intranasally with 6 mL of tissue culture supernatant containing 1.0 × 10^4^ tissue culture infective dose of 50% (TCID_50_)/mL of type 1 PRRSV (SNUVR090485, 2^nd^ passage in alveolar macrophages). The vaccinated sows from group 2 and the non-vaccinated sows from group 4 were inoculated intranasally with 6 mL of tissue culture supernatant containing 1.0 × 10^4^ TCID_50_/mL of type 2 PRRSV (SNUVR100059, 2^nd^ passage in MARC-145 cells). The vaccinated sows from group 5 and the negative control sows from group 6 were similarly inoculated with uninfected cell culture supernatant. Each inoculum was instilled over a period of 4–5 min into both nostrils. The sows were housed in isolation facilities and allowed to farrow naturally, under supervision. Blood samples from each sow were collected by jugular venipuncture at -28, -21, 0, 56, 93, 100, 107, and 114 days of gestation.

All live-born piglets were humanely euthanized with an intravenous overdose of pentobarbital for tissue collection and evaluation. All expelled fetuses (mummified, dead, and live-born) from all study groups were necropsied and evaluated for gross lesions. Crown-to-rump measurements were used to determine the approximate gestational time of fetal death for the mummified and dead fetuses [15]. This study was approved by the Seoul National University Institutional Animal Care and Use Committee.

### Serology

The serum samples were tested using the commercially available PRRSV ELISA (HerdCheck PRRS 2XR, IDEXX Laboratories Inc., Westbrook, ME, USA). Serum virus neutralization (SVN) tests were also performed using a heterologous challenging type 1 or type 2 PRRSV. Serum samples were heat-inactivated for 45 min at 56 °C before testing. Each serum was then diluted using a twofold serial dilution technique in RPMI-1640 (Sigma Aldrich Corporation, St. Louis, MO, USA) supplemented with 10% FCS (Sigma), 20 mM L-glutamine (Cellgro, Herdon, VA, USA), and an antibiotic–anti-mycotic mixture (Sigma Aldrich Corporation) which consisted of 100 IU/mL penicillin, 100 mg/mL streptomycin, 50 mg/mL gentamicin, and 0.25 mg/mL amphotericin B (here-after, RPMI growth medium). One hundred microliters of each diluted sample was mixed with an equal volume of each virus at a rate of 1.0 × 10^3^ TCID_50_/mL of both a heterologous challenging type 1 and type 2 PRRSV. Mixtures were incubated for 1 h at 37 °C and then each mixture was inoculated onto MARC-145 cell mono-layers prepared in 96-well Plates 24 h earlier. Each sample was run in duplicate. After 1h incubation at 37 °C, all inocula were removed and replaced with 200 μL of RPMI growth medium. Thereafter, the cells were incubated at 37 °C and monitored daily for cytopathic effect (CPE). The titer of inoculated virus was verified by the back titration of the inoculum. The presence of virus-specific CPE in each well was recorded after incubating for 7 days. The presence of virus in wells without CPE was further determined by immunofluorescence microscopy using SDOW17-FITC conjugate (Rural Technologies Inc., Brookings, SD, USA) [16,17]. Serum samples were considered to be positive for PRRSV neutralizing antibodies (NA) if the titer was greater than 2.0 (log_2_) [18].

### Enzyme-linked immunospot (ELISPOT) assay

The numbers of PRRSV-specific interferon-γ-secreting cells (IFN-γ-SC) were determined in peripheral blood mononuclear cells (PBMC) at -28, -21, 0, 56, 93, 100, 107 and 114 days of gestation as previously described [19,20] with some modifications. Briefly, 50 μL containing 5 × 10^5^ PBMC in RPMI 1640 medium that was supplemented with 10% fetal bovine serum (HyClone Laboratories, Inc., SelectScience, Bath, UK), 1 mM non-essential amino acids (Invitrogen, Carlsbad, CA, USA), 1 mM sodium pyruvate, 5 mM 2-mercaptoethanol, 50 000 IU/l penicillin l, and 50 mg/L streptomycin were seeded into plates that were precoated overnight at 4 °C with anti-porcine IFN-γ monoclonal antibody (10 μg/mL, MABTECH, Mariemont, OH, USA). The plates were stimulated by addition of either a heterologous challenging type 1 or type 2 PRRSV solution in RPMI 1640 medium for 20 h at 37 °C in a 5% humidified CO_2_ atmosphere. The linear response was tested between 0.1 and 1 MOI (multiplicity of infection). Phytohemagglutinin (10 μg/mL, Roche Diagnostics GmbH, Mannheim, Germany) and culture medium were used as positive and negative controls, respectively. Next, the wells were washed five times with PBS (200 μL per well). Thereafter, the procedure was conducted according to the manufacturer’s instructions using the commercial ELISPOT Assay Kit (MABTECH). The spots on the membranes were read by an automated ELISPOT Reader (AID ELISPOT Reader, AID GmbH, Strassberg, Germany). The results were expressed as the numbers of IFN-γ-SC per million PBMC.

### Quantification of PRRSV RNA in blood

RNA extractions from the serum samples were performed as previously described [21,22]. Real-time PCR for type 1 and type 2 PRRSV, and vaccine strain were used to quantify PRRSV genomic cDNA copy numbers using RNA extraction from serum samples. The sequences of primers and probes in real-time PCR are 100% complementary to the sequences of the challenge viruses (except 89.5% complementary for reverse primer of type 2 PRRSV). Real-time PCR was considered positive if the cycle threshold (*C*_*T*_) level was obtained at ≤ 45 cycles.

To construct a standard curve, real-time PCR was performed in quadruplicate in two different assays: (i) 10-fold serial dilutions of the PRRSV plasmid were used as the standard, with concentrations ranging from 10^10^ to 10^3^ copies/mL and (ii) 10-fold serial dilutions of the challenging type 1 and type 2 PRRSV cultured in alveolar macrophages and MARC-145 cells, respectively, from 1.0 × 10^6^ TCID_50_/mL to 1.0 × 10^-1^ TCID_50_/mL. The PRRSV plasmid was prepared as previously described [23]. Briefly, the transcript cDNA product was cloned into the pCR2.1 plasmid (Invitrogen, Carlsbad, CA, USA). The recombinant plasmid was purified using a plasmid Miniprep kit (Qiagen, Valencia, CA, USA) according to the manufacturer’s instructions, and the concentration of the purified plasmid was determined using a spectrophotometer.

### Virus isolation and sequence analysis

PRRSV was isolated from live-born piglets and stillborn fetuses as previously described [24]. The isolated PRRSV from fetuses were further analyzed for the ORF5 sequence. Sequencing was performed on the purified RT-PCR products of amplified ORF5 [24,25].

### In situ hybridization

The probe for the type 1 and type 2 PRRSV was generated from challenging type 1 and type 2 PRRSV by PCR [26]. In situ hybridization (ISH) for the detection of type 1 and type 2 PRRSV nucleic acid in fetal tissues was performed and analyzed morphometrically as previously described [26,27].

### Statistical analysis

ANOVA with a post-hoc Tukey’s test was used to compare the primary variables for single comparison (ELISA and SVN test, IFN-γ-SC, and PRRSV RNA quantification) among the sows in 4 groups (groups 1, 2, 3, and 4). ANOVA with a post-hoc Tukey’s test was used to compare the primary variables (ISH scores) across the litters of sows from the 4 groups (groups 1, 2, 3, and 4). The Pearson’s correlation coefficient was used to assess the relationship among viremia, and PRRSV-specific NA titers and IFN-γ-SC. *P* < 0.05 indicated statistical significance.

## Results

### Pregnancy

Sows in groups 1, 3, and 4 farrowed between 102 and 109 days of gestation while sows in groups 2, 5, and 6 carried their pregnancies to term and farrowed between 114 and 115 days of gestation. The number of litters from the sows in all 6 groups is summarized in Table 2.

**Table 2:** Litter characteristics at parturition from sows in the 6 groups

**Group ( *n *)**^**a**^	**Gestation**	**Litter characteristics**			
			***n***	**Average length**^**b **^**(cm)**	**Length range**^**b **^**(cm)**
1 (5)	106-109	Live-born	3	29.4	29.0-30.1
		Stillborn	45	28.8	28.1-29.6
		Mummified	1	14.1	13.4-16.3
2 (5)	Term	Live-born	49	30.5	30.1-32.4
		Stillborn	2	29.2	29.0-29.4
		Mummified	-	-	-
3 (5)	102-107	Live-born	5	28.9	28.6-29.4
		Stillborn	42	27.9	27.1-28.5
		Mummified	-	-	-
4 (5)	103-108	Live-born	7	27.9	27.6-29.1
		Stillborn	41	27.3	27.1-27.7
		Mummified	-	-	-
5 (3)	Term	Live-born	26	31.3	30.8-32.1
		Stillborn	1	29.5	29.5
		Mummified	-	-	-
6 (3)	Term	Live-born	29	31.6	30.3-32.1
		Stillborn	-	-	-
		Mummified	-	-	-

^a^*n*, number of sows in group.

^b^Estimation of fetal death based on crown-to-rump length.

### Anti- PRRSV IgG antibodies in sows

**Figure 1:**
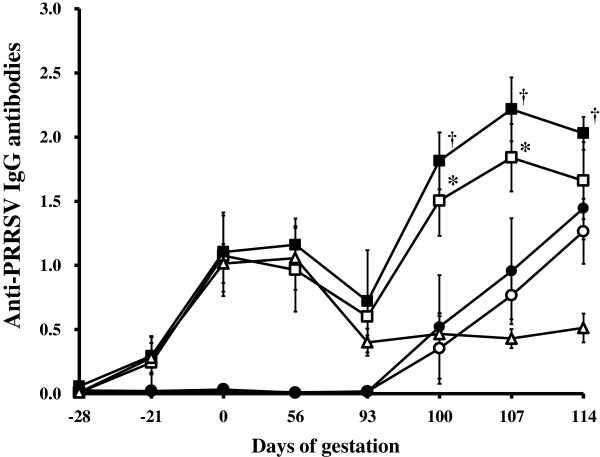
**Mean group anti-PRRSV IgG antibody response in different treatment groups.** Sows that received PRRSV vaccine followed by type 1 PRRSV challenge (group 1, □); sows that received PRRSV vaccine followed by type 2 PRRSV challenge (group 2, ■); non-vaccinated sows challenged with type 1 PRRSV (group 3, ○); non-vaccinated sows challenged with type 2 PRRSV (group 4, ●); and sow that received PRRSV vaccine (group 5, △). Variation is expressed as the standard deviation. ^*^Significant (*P* < 0.05) difference between group 1 and group 3. ^†^Significant (*P* < 0.05) difference between group 2 and group 4.

The IgG antibody response in sows was measured using a commercially available ELISA. The results from these experiments are summarized in Figure 1. Anti-PRRSV IgG antibodies were not detected in the serum samples at -28 days of gestation (time to PRRSV vaccination) in sows from any of the 6 groups but were detected in the serum samples at days 0 (28 days post-vaccination), 56, and 93 (time to PRRSV challenge) of gestation in sows that received the PRRSV vaccine (groups 1, 2, and 5). At days 100 and 107 of gestation, sows that received the PRRSV vaccine followed by type 1 PRRSV challenge (group 1) and type 2 PRRSV challenge (group 2) had significantly higher anti-PRRSV IgG antibody levels (*P* < 0.05) than non-vaccinated sows challenged with either type 1 PRRSV (group 3) or type 2 PRRSV (group 4), respectively. At day 114 of gestation, the sows that had received the PRRSV vaccine followed by the type 2 PRRSV challenge (group 2) had significantly higher anti-PRRSV IgG antibody levels (*P* < 0.05) than the non-vaccinated sows in group 4 (Figure 1).

### PRRSV-specific neutralizing antibodies

Serum virus neutralization (SVN) tests were also performed using both type 1 and type 2 PRRSV. The results are summarized in Table 3. No type 1 or type 2 PRRSV-specific NA titers were detected in sera from sows in 4 groups (groups 1, 2, 3, and 4) at -28, -21, 0, 56, and 93 days of gestation. After challenge with type 1 PRRSV, sera from sows that had received type 2 PRRSV-based vaccine followed by type 1 challenge (group 1) showed similar neutralizing activity against type 1 and type 2 PRRSV at 100, 107, and 114 days of gestation. In contrast, sera from sows that had received the type 2 PRRSV-based vaccine followed by type 2 PRRSV challenge (group 2) showed significantly higher neutralizing activity against type 2 PRRSV than that against type 1 PRRSV at 100 (*P* = 0.004), 107 (*P* = 0.001), and 114 (*P* = 0.001) days of gestation. Sera from non-vaccinated sows that had been challenged with type 1 PRRSV (group 3) showed similar neutralizing activity against type 1 and type 2 PRRSV at 100, 107, and 114 days of gestation. Sera from non-vaccinated sows that had been challenged with type 2 PRRSV (group 4) showed significantly higher neutralizing activity against type 2 PRRSV than against type 1 PRRSV at 107 (*P* = 0.006) and 114 (*P* = 0.004) days of gestation (Table 3). No type 1 and type 2 PRRSV-specific NA titer was detected in the 2 control groups that were not challenged with PRRSV (groups 5 and 6).

**Table 3:** Serum viral neutralization test results using type 1 and type 2 PRRSV

	**Against type 1 PRRSV**				**Against type 2 PRRSV**			
	**Days of gestation**				**Days of gestation**			
**Group**	**93**	**100**	**107**	**114**	**93**	**100**	**107**	**114**
1	0/5^*^	0/5	2/5^a^	3/5	0/5	2/5	3/5	5/5
	0.40 ± 0.79	1.73 ± 0.85	2.29 ± 0.41^b^	2.21 ± 0.19	0.25 ± 0.50	2.11 ± 0.19	2.26 ± 0.49	2.75 ± 0.50
2	0/5	0/5	0/5	1/5	0/5	3/5	4/5	5/5
	0.25 ± 0.50	0.75 ± 0.50	1.29 ± 0.34	2.05 ± 0.07^c^	0.25 ± 0.50	2.34 ± 0.46^*^	3.51 ± 0.56^*^	3.75 ± 0.50^*^
3	0/5	0/5	2/5	3/5	0/5	1/5	1/5	2/5
	-	1.15 ± 0.29	2.01 ± 0.02	2.19 ± 0.34	-	2.02 ± 0.02	2.01 ± 0.01	2.11 ± 0.18
4	0/5	0/5	0/5	1/5	0/5	1/5	2/5	4/5
	-	0.50 ± 0.58	1.15 ± 0.29	2.02 ± 0.02	-	2.01 ± 0.01	2.00 ± 0.01^*^	2.97 ± 0.53^*^

^a^Number of positive/negative sows.

^b^Mean titers (log_2_) and standard deviation. Mean titers have been calculated as the arithmetic mean of positive sera only.

^c^Repeated value of single positive sample.

^*^Statistical difference (*P* < 0.05) for neutralizing activity against type 1 PRRSV and against type 2 PRRSV at the same days of gestation.

### PRRSV-specific interferon- γ-secreting cells

**Figure 2:**
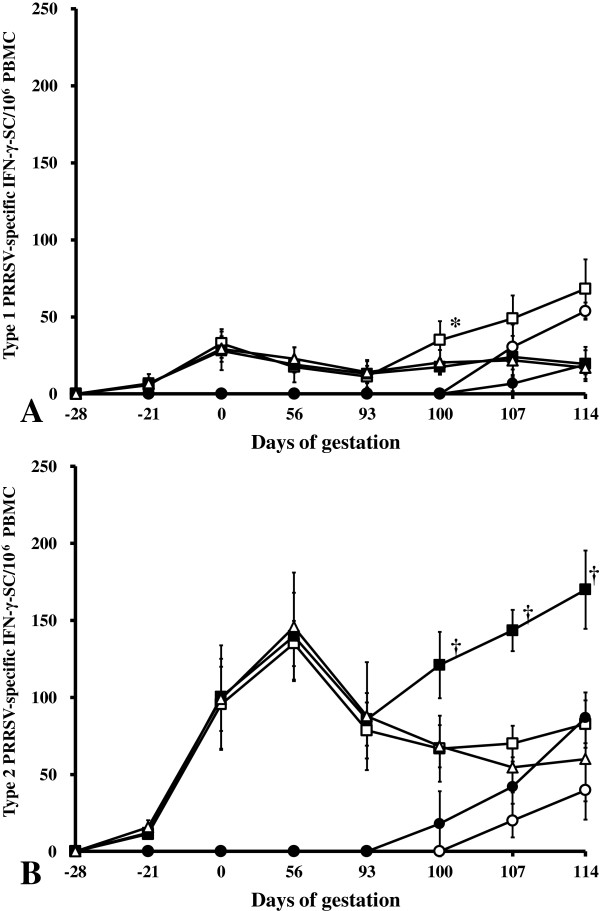
**Frequency of type 1 (A) and type 2 (B) PRRSV-specific IFN-γ-SC/10**^**6 **^**PBMC.** Sows that received PRRSV vaccine followed by type 1 PRRSV challenge (group 1, □); sows that received PRRSV vaccine followed by type 2 PRRSV challenge (group 2, ■); non-vaccinated sows challenged with type 1 PRRSV (group 3, ○); non-vaccinated sows challenged with type 2 PRRSV (group 4, ●); and sows that received PRRSV vaccine (group 5, △).^*^Significant (*P* < 0.05) difference between group 1 and group 3. ^†^Significant (*P* < 0.05) difference between group 2 and group 4.

To further access the immunological response to PRRSV challenge, PRRSV-specific IFN-γ-SC were measured. The results are summarized in Figure 2. When PBMC were stimulated with type 1 PRRSV, mean frequencies of type 1 PRRSV-specific IFN-γ-SC remained at basal levels (< 20 cells/10^6^ PBMCs) in sows from 5 groups at -28, 0, 56, and 93 days of gestation. At later times, the frequency of type 1 PRRSV-specific IFN-γ-SC began to increase and reached an average of 68.3 ± 19.1 cells/10^6^ PBMC in sows having received the type 2 PRRSV-based vaccine followed by type 1 PRRSV challenge (group 1) at 114 days of gestation (21 days post challenge) and 53.8 ± 5.6 cells/10^6^ PBMC in non-vaccinated sows that had been challenged with type 1 PRRSV challenge (group 3; Figure 2A). Mean frequencies of type 1 PRRSV-specific IFN-γ-SC remained at basal levels (< 20 type 1 PRRSV-specific IFN-γ-SC/10^6^ PBMC) in sows from the 3 groups (groups 2, 4, and 5) at 100, 107, and 114 days of gestation.

When PBMC were stimulated with type 2 PRRSV, mean frequencies of type 2 PRRSV-specific IFN-γ-SC remained at basal levels (< 20 cells/10^6^ PBMC) in sows that had received the type 2 PRRSV-based vaccine followed by type 1 PRRSV challenge (group 1) and by type 2 PRRSV challenge (group 2) at -28 and -21 days of gestation. At later time points, the frequency of type 2 PRRSV-specific IFN-γ-SC/10^6^ PBMC began to increase and reached an average of 144.0 ± 30.6 cells/10^6^ PBMC in sows (groups 1, 2, and 5) at 56 days of gestation and decreased an average of 85.3 ± 21.5 cells/10^6^ PBMC in sows (groups 1, 2, and 5) at 93 days of gestation. Upon challenging with PRRSV, while mean frequencies was further enhanced to 170.0 ± 25.4 cells/10^6^ PBMC in sows (group 2), mean frequencies did not show any significant change in sows (group 1), and decreased gradually in non-challenged sows (group 5) at 114 days of gestation. Mean frequencies of type 2 PRRSV-specific IFN-γ-SC remained at basal levels (< 20 type 2 PRRSV-specific IFN-γ-SC/10^6^ PBMC) in sows that had been challenged with type 1 PRRSV (group 3) and type 2 PRRSV (group 4) at -28, -21, 0, 56, and 93 days of gestation. At later times, the frequency of type 2 PRRSV-specific IFN-γ-SC/10^6^ PBMC began to increase and reached an average of 39.8 ± 19.1 cells/10^6^ PBMC in sows (group 3) and 86.8 ± 16.5 cells/10^6^ PBMC (group 4) at 114 days of gestation (Figure 2B). No type 1 and type 2 PRRSV-specific IFN-γ-SC was detected in negative control sows (group 6) throughout the experiment.

When equivalent series of IFN-γ-ELISPOT results were compared between type 1 PRRSV stimulation versus type 2 PRRSV stimulation within the same groups, some differences were seen (Figures 2A and B). Thus, stimulation with type 2 PRRSV produced higher frequencies of IFN-γ-SC at 0, 56, 93 days of gestation compared to the stimulation with type 1 PRRSV (95.5 ± 29.6 vs. 32.7 ± 9.4, 135.0 ± 14.6 vs. 17.5 ± 3.7, 78.5 ± 18.2 vs. 11.3 ± 7.1, respectively) in sows that had received the type 2 PRRSV-based vaccine followed by type 1 PRRSV challenge (group 1). After 14 days post challenge of type 1 PRRSV in group 1, there were no significantly different frequencies of IFN-γ-SC between stimulation with type 1 and type 2 PRRSV. In contrast, stimulation with type 2 PRRSV produced higher frequencies of IFN-γ-SC at 0, 56, 93, 100, 107, 114 days of gestation compared to stimulation with type 1 PRRSV (103.5 ± 33.5 vs. 28.0 ± 12.5, 139.8 ± 28.1 vs. 19.0 ± 11.4, 85.8 ± 17.1 vs. 13.0 ± 9.1, 121.7 ± 21.6 vs. 17.5 ± 4.2, 143.5 ± 13.4 vs. 24.0 ± 13.7, 170.0 ± 25.4 vs. 19.5 ± 11.1, respectively) in sows that had received type 2 PRRSV-based vaccine followed by type 2 PRRSV challenge (group 2).

### Quantification of PRRSV RNA in sow sera

The amount of PRRSV RNA in the sera of sows was quantified using RT-PCR. Standard curves were constructed by plotting the ten-fold serial diluted plasmid copy number logarithm against the measured *C*_*T*_ values. The linear correlation (*R*^*2*^) between the *C*_*T*_ and the plasmid copy number logarithm were repeatedly greater than 0.998 for the type 1 PRRSV and 0.997 for the type 2 PRRSV. The detection limit of the real-time PCR was shown to be equivalent to 37 type 1 PRRSV copies (0.63 log_10_)/reaction and 41 type 2 PRRSV copies (0.62 log_10_)/reaction.

**Figure 3:**
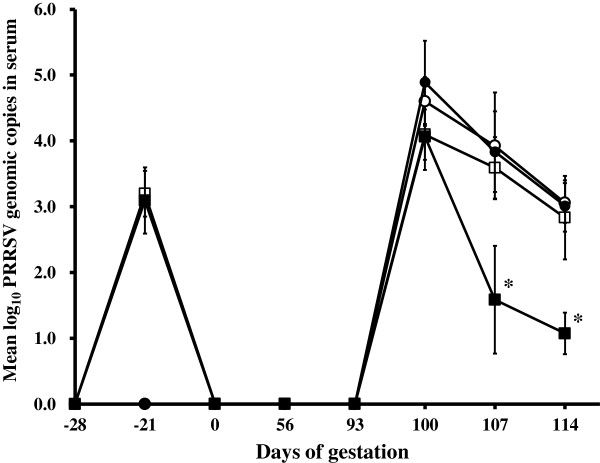
**Mean values of the genomic copies of PRRSV RNA in serum in different treatment groups.** Sows that received PRRSV vaccine followed by type 1 PRRSV challenge (group 1, □); sows that received PRRSV vaccine followed by type 2 PRRSV challenge (group 2, ■); non-vaccinated sows challenged with type 1 PRRSV (group 3, ○); and non-vaccinated sows challenged with type 2 PRRSV (group 4, ●). ^*^Significant (*P* < 0.05) difference between group 2 and group 4.

Genomic copies of the vaccine strain were detected in the serum of the vaccinated sows (groups 1, 2, and 5) at -21 days of gestation. Thereafter, no vaccine strain was detected in the serum from vaccinated sows (Figure 3). Genomic copies of type 1 PRRSV were detected in the serum of the sows that had received type 2 PRRSV-based vaccine followed by the type 1 PRRSV challenge (group 1) and of the non-vaccinated sows challenged with type 1 PRRSV (group 3) at days 100, 107, and 114 of gestation. However, there were no significant differences between groups 1 (vaccinated) and 3 (non-vaccinated) in terms of their genomic copy number of type 1 PRRSV at days 100, 107, and 114 of gestation (Figure 3). Genomic copies of type 2 PRRSV were detected in the serum of the sows that had received type 2 PRRSV-based vaccine followed by the type 2 PRRSV challenge (group 2) and of the non-vaccinated sows that had been challenged with the type 2 PRRSV (group 4) at days 100, 107, and 114 of gestation. The non-vaccinated sows in group 4 had significantly higher genomic copies of type 2 PRRSV in their serum at gestational days 107 (*P* = 0.002) and 114 (*P* < 0.001) than did the vaccinated sows in group 2 (Figure 3). No PRRSV or vaccine strain was detected in the serum of the negative control sows throughout the experiment.

The number of genomic copies of type 2 PRRSV in the blood correlated inversely with type 2 PRRSV-specific NA titers (*r*^2^ = -0.998, *P* = 0.003) and type 2 PRRSV-specific IFN-γ-SC (*r*^2^ = -0.996, *P* = 0.050) in sows that had received type 2 PRRSV-based PRRSV vaccine followed by type 2 challenge (group 2).

### Quantification of PRRSV RNA in stillborn fetuses and live-born piglets

The amount of PRRSV RNA was also quantified in the tissues of stillborn and live-born piglets. Litters from non-vaccinated sows challenged with type 2 PRRSV (group 4) had significantly higher scores for the mean number of PRRSV-positive cells per unit area in several organs (*P* < 0.05) than those from sows that received type 2 PRRSV-based vaccine followed by type 2 PRRSV challenge (group 2). No PRRSV or vaccine strain was detected in several organs from sows in the 2 control groups (groups 5 and 6) (Table 4).

**Table 4:** Virus isolation, and mean scores for PRRSV-positive cells by in situ hybridization (ISH) and real-time PCR-positive numbers in litters from sows

**Test**	**Group**	***n***	**Lung**	**LN**^**a**^	**Heart**	**Tonsil**	**Thymus**	**Liver**	**Spleen**
PRRSV	1	48	41^b^	39	31	40	43	18	30
(real-time PCR)			(1.3 ± 0.3)^c^	(1.5 ± 0.4)	(0.4 ± 0.5)	(1.4 ± 0.6)	(2.9 ± 0.6)	(0.3 ± 0.3)	(1.1 ± 0.4)
	2	51	6	5	4	4	7	0	4
			(0.3 ± 0.1)^*^	(0.4 ± 0.2)^*^	(0.1 ± 0.3)^*^	(0.4 ± 0.3)^*^	(0.5 ± 0.3)^*^	0^*^	(0.2 ± 0.3)^*^
	3	47	44	41	32	42	44	21	33
			(1.5 ± 0.5)	(1.8 ± 0.5)	(0.5 ± 0.4)	(1.5 ± 0.6)	(3.2 ± 0.7)	(0.3 ± 0.1)	(1.0 ± 0.5)
	4	48	42	40	28	39	45	19	35
			(1.4 ± 0.2)	(1.5 ± 0.7)	(0.2 ± 0.2)	(1.1 ± 0.3)	(2.8 ± 0.4)	(0.3 ± 0.3)	(0.9 ± 0.4)
	5	27	0	0	0	0	0	0	0
	6	29	0	0	0	0	0	0	0
PRRSV	1	48	5.7 ± 1.9	13.5 ± 3.7	4.0 ± 0.9	13.1 ± 3.5	31.5 ± 6.9	2.4 ± 0.5	13.7 ± 2.3
(ISH)	2	51	2.4 ± 0.9^*^	4.5 ± 1.1^*^	0.4 ± 0.5^*^	4.5 ± 1.2^*^	6.5 ± 2.4^*^	0^*^	1.6 ± 0.7^*^
	3	47	7.3 ± 1.7	16.7 ± 3.9	3.4 ± 1.2	15.2 ± 2.9	40.8 ± 4.8	1.9 ± 0.8	13.9 ± 2.4
	4	48	7.1 ± 2.2	14.8 ± 1.9	2.6 ± 1.3	15.0 ± 3.4	39.9 ± 5.1	1.8 ± 0.7	13.5 ± 2.6
	5	27	0	0	0	0	0	0	0
	6	29	0	0	0	0	0	0	0
PRRSV	1	48	31	33	20	30	34	11	23
(Isolation)	2	51	0	0	0	0	0	0	0
	3	47	34	36	21	29	37	14	27
	4	48	33	35	20	26	35	12	27
	5	27	0	0	0	0	0	0	0
	6	29	0	0	0	0	0	0	0

^a^LN, inguinal lymph node.

^b^Number of positive fetuses.

^c^Mean values of the genomic copies of PRRSV RNA in fetal tissues (log_10_).

^*^Significant (*P* < 0.05) difference between group 2 and group 4.

### In situ hybridization

**Figure 4:**
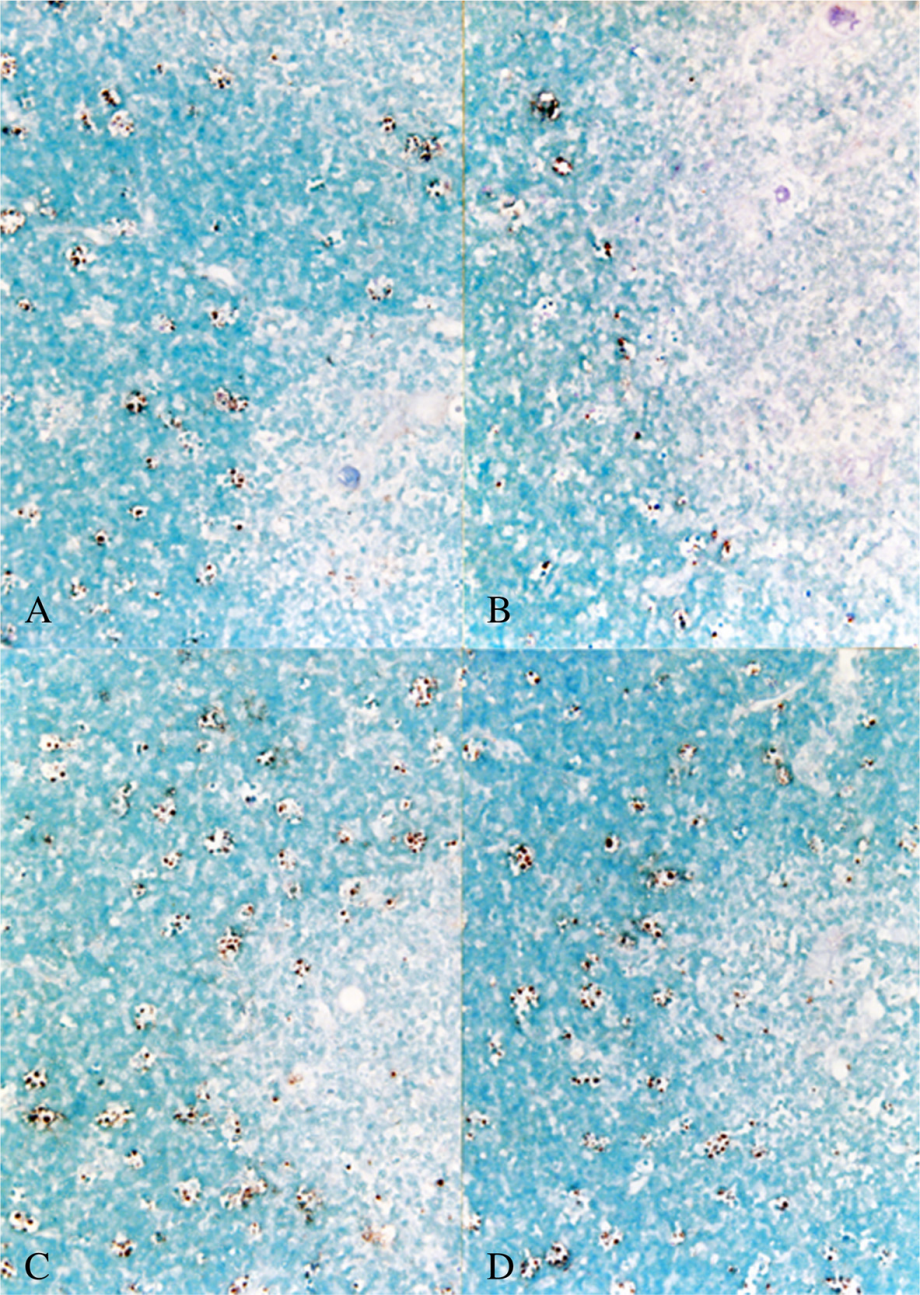
**In situ hybridization for the detection of PRRSV nucleic acid in the thymuses of the litters.** The results are the sows that received PRRSV vaccine followed by type 1 PRRSV challenge (group 1, **A)**; sows that received PRRSV vaccine followed by type 2 PRRSV challenge (group 2, **B)**; sows challenged with type 1 PRRSV (group 3, **C)**; sows challenged with type 2 PRRSV (group 4, **D)**.

In situ hybridization was also performed to detect type 1 and type 2 PRRSV genomes in the tissues from piglets. As expected, type 1 PRRSV-positive cells were only detected in the litters of piglets from sows in groups 1 and 3, while type 2 PRRSV-positive cells were only detected in the litters from sows in groups 2 and 4 (Table 4). No type 1 or type 2 PRRSV was detected in any litters from sows in control groups 5 and 6. Regardless of their PRRSV genotype, the positive cells generally had large oval nuclei and abundant cytoplasm, which was consistent with the morphology of macrophages in several organs (Figure 4).

The score for the mean number of PRRSV-positive cells per unit area of the organs examined did not differ significantly between the litters of sows that had received type 2 PRRSV vaccine followed by the type 1 PRRSV challenge (group 1) and sows that had been challenged with type 1 PRRSV (group 3) (Table 4). Litters from non-vaccinated sows challenged with type 2 PRRSV (group 4) had significantly higher scores for the mean number of PRRSV-positive cells per unit area in several organs (*P* < 0.05) than those from sows that received type 2 PRRSV-based vaccine prior to type 2 PRRSV challenge (group 2) (Table 4).

### Virus isolation and sequence analysis

Type 1 and type 2 PRRSV were isolated from several organs from the live-born piglets and stillborn fetuses from sows in groups 1, 2, 3, and 4 (Table 4). All isolated type 1 and type 2 PRRSV strains were confirmed by sequence analysis to be of the same propagating virus as the challenge stock. No PRRSV was isolated from the serum of sows in control groups 5 or 6. Vaccine strains were not isolated from the live-born piglets or stillborn fetuses from any sows used in this study.

## Discussion

This study clearly demonstrates that the vaccination of sows with the commercial type 2 PRRSV-based vaccine protects pregnant sows against heterologous type 2 PRRSV challenge but not against type 1 PRRSV challenge. The statistical analysis shows that the vaccine elicited a significant improvement in the number of live-born pigs and a decrease in the number of mummified fetuses in vaccinated sows challenged with the heterologous type 2 PRRSV (Table 2). Analysis of crown-to-rump length is a critical parameter for the evaluation of the effect of vaccination. Since crown-to-rump length of stillborn fetuses was not significantly different between sows vaccinated against type 2 PRRSV challenge and negative control sows, these data further supported the protection of type 2 PRRSV-based vaccine against type 2 PRRSV challenge.

Our results were in agreement with the previous findings where the type 2 PRRSV-based vaccine provided only partial protection against subsequent challenge of heterologous virulent type 2 PRRSV in pregnant sows [28,29]. Moreover, vaccination with type 1 PRRSV-based vaccine reduced the level of viremia and clinical signs after challenge with type 1 PRRSV, but barely reduced the level of viremia and clinical signs after challenge with the type 2 PRRSV challenge in preweaning pigs and vice versa [30,31]. However, a recent cross-protection study has found that immunization with a type 1 PRRSV-based vaccine provides partial protection against challenge with a highly virulent type 2 PRRSV [32]. Therefore, the extent of cross-protection is not solely related to the genomic differences between the two genotypes.

Virological evaluation demonstrated that the type 2 PRRSV-based vaccine reduced the type 2 PRRSV load but not the type 1 PRRSV load in the sow blood (Figure 3). Because PRRSV was detected in the litters of pregnant sows infected intranasally with PRRSV, PRRSV can infect pregnant sows and can be transmitted from sow to fetus through viremia and transplacental infection. Hence, PRRSV in the blood plays an important role in the dissemination of PRRSV to the fetuses of the pregnant sows. Vaccination can decrease the PRRSV load in the blood of pregnant sows and can subsequently decrease the risk of maternal to fetal transmission through viremia. However, sows that received a type 2 PRRSV-based vaccine followed by a type 2 PRRSV challenge (group 2) were also viremic similarly to any other group at early time points. Reduction of viremia was not clearly seen until 107 days of gestation. Thus, late abortions may have been prevented by the reduction of viremia; however, this link is not as clear with early abortions, since at day 100 all sows were equally viremic. Further studies are needed to elucidate the protective role of the PRRSV vaccine at preventing early abortion in sows that received a type 2 PRRSV-based vaccine followed by a type 2 PRRSV challenge.

For the pathological evaluation, the detection of PRRSV replication within fetal tissues is critical in order to determine the efficacy of the PRRSV vaccines. In the present study, the vaccination of pregnant sows with the type 2 PRRSV-based vaccine effectively reduced the level of type 2 PRRSV nucleic acids and cell death in the fetal tissues of litters from vaccinated and type 1 PRRSV-challenged sows. However, vaccination with the type 2 PRRSV-based vaccine does not reduce the level of type 1 PRRSV nucleic acid in the fetal tissues of litters from the vaccinated and type 2 PRRSV-challenged sows.

Upon PRRSV challenge at 93 days of gestation (121 days post vaccination), NA titers were detected in vaccinated-challenged sows. Interestingly, when cross-neutralization assays were performed, the NA titers against type 2 PRRSV were significantly higher than the NA titers against type 1 PRRSV in the sera of sows that had received a type 2 PRRSV-based vaccine and a subsequent type 2 PRRSV challenge. Our results provide indirect evidence that the reactivity is not the same for type 1 PRRSV antibodies against type 2 PRRSV as for type 2 PRRSV antibodies against type 1 PRRSV. These observations are further supported by previous studies in which the inoculation with different PRRSV strains resulted in varying levels of NA titers against the PRRSV strains by cross-neutralization assays [33,34]. In addition, homologous NA plays an important role in protection against experimental challenge and protection is NA titers dependent [35,36]. Although there was a correlation between NA titers and PRRSV viremia in vaccinated and challenged pigs, its precise role in the clearance of viremia is uncertain because the NA levels were low in the vaccinated and vaccinated-challenged sows throughout the experiment. However, this study was not evaluating protection conferred by the vaccine by measuring viability not only at birth but also at weaning, as has often been done by previous publications using a reproductive failure model [35,37]. Further studies are needed to determine the protective role of NA in newborn piglets from vaccinated and challenged sows during the postnatal period.

Despite the low NA levels in response to PRRSV vaccination [38], the sows that received the type 2 PRRSV-based vaccine followed by a type 2 PRRSV challenge still efficiently cleared type 2 PRRSV in the blood. Presumably this was at least partially dependent on cell-mediated immunity, especially the host IFN-γ response. IFN-γ is known to inhibit PRRSV replication [39,40]. The sows that received a type 2 PRRSV-based vaccine had significantly higher frequencies of type 2 PRRSV-specific IFN-γ-SC than frequencies of type 1 PRRSV-specific IFN-γ-SC. The frequency of type 2 PRRSV-specific IFN-γ-SC was approximately 75 PRRSV-specific IFN-γ-SC/10^6^ PBMC at the day of challenge. The vaccinated sows that did not develop viremia had 45 PRRSV-specific IFN-γ-SC/10^6^ PBMC at the days of challenge [41]. Therefore, the 75 PRRSV-specific IFN-γ-SC/10^6^ PBMC were sufficient to reduce or prevent type 2 PRRSV viremia after challenge with the virulent type 2 PRRSV in sows that had received a type 2 PRRSV-based vaccine. Our results are further supported by previous studies in which infections with different PRRSV strains led to different PRRSV-specific IFN-γ-SC outcomes, resulting in different degrees of heterologous protection [33,42]. In addition, there is a strong correlation between cell-mediated immunity, as measured by IFN-γ-SC, and protection against reproductive failure in sows [43]. A positive correlation between type 2 PRRSV-specific IFN-γ-SC and type 2 PRRSV viremia was found in sows that received a type 2 PRRSV-based vaccine followed by a type 2 PRRSV challenge. These results strongly suggest that PRRSV-specific IFN-γ-SC is likely the main factor in the protection of pregnant gilts against PRRSV. Therefore, differences in the induction of PRRSV-specific IFN-γ-SC by type 2 PRRSV-based vaccine against challenge by type 1 and type 2 PRRSV may contribute to different protective outcomes. Alternatively, it cannot be ruled out that levels of protection in vaccinated sows may be directly due to using a different challenge strain (type 1 vs. type 2 virus) regardless of different frequency of PRRSV-specific IFN-γ-SC.

The vaccine company claims that the duration of immunity is throughout the gestation periods or at least 4 months post-vaccination [44]. Since vaccinated sows were challenged with PRRSV at 3 weeks antepartum, antigenic differences rather than duration of immunity could be a major factor that influenced the outcome. The results of this study indicate that sows that had received type 2 PRRSV-based vaccine differ in their response to PRRSV-specific neutralization and IFN-γ-SC against different challenging type 1 and type 2 PRRSV strains. Low efficiency of the type 2 PRRSV-based vaccine against type 1 PRRSV is most likely due to the antigenic differences between the vaccine and the challenge virus.

## Competing interests

The authors declare that they have no competing interests.

## Authors’ contributions

KH and HWS performed the experimental trials, data analysis and writing of the manuscript, CP prepared the inocula and lab analysis and inoculation of virus, CC development of protocol, design of the study, review of the final manuscript, approval for publication. All authors read and approved the final manuscript.

## References

[B1] Zimmerman JJ Benfield DA Dee SA Murtaugh MP Stadejek T Stevenson GW Torremorell M Zimmerman JJ, Karriker LA, Ramirez A, Schwartz KJ, Stevenson GW Porcine reproductive and respiratory syndrome virus (porcine arterivirus) Diseases of Swine 2012 10 Ames: Wiley-Blackwell Publishing 461 486

[B2] Nelsen CJ Murtaugh MP Faaberg KS Porcine reproductive and respiratory syndrome virus comparison: divergent evolution on two continents J Virol 1999 73 270 280 9847330 10.1128/jvi.73.1.270-280.1999 PMC103831

[B3] Allende R Lewis TL Lu Z Rock DL Kutish GF Ali A Doster AR Osorio FA North American and European porcine reproductive and respiratory syndrome viruses differ in non-structural protein coding regions J Gen Virol 1999 80 307 315 10073689 10.1099/0022-1317-80-2-307

[B4] Murtaugh MP Stadejek T Abrahante JE Lam TT Leung FC The ever-expanding diversity of porcine reproductive and respiratory syndrome virus Virus Res 2010 154 18 30 10.1016/j.virusres.2010.08.015 20801173

[B5] Mateu E Diaz I The challenge of PRRS immunology Vet J 2008 177 345 351 10.1016/j.tvjl.2007.05.022 17644436 PMC7110845

[B6] Nelson EA Christopher-Hennings J Drew T Wensvoort G Collins JE Benfield DA Differentiation of U.S. and European isolates of porcine reproductive and respiratory syndrome virus by monoclonal antibodies J Clin Microbiol 1993 31 3184 3189 7508455 10.1128/jcm.31.12.3184-3189.1993 PMC266373

[B7] Labarque G Van Reeth K Nauwynck H Drexler C Van Gucht S Pensaert M Impact of genetic diversity of European-type porcine reproductive and respiratory syndrome virus strains on vaccine efficacy Vaccine 2004 22 4183 4190 10.1016/j.vaccine.2004.05.008 15474708

[B8] Kapur V Elam MR Pawlovich TM Murtaugh MP Genetic variation in porcine reproductive and respiratory syndrome virus isolates in the midwestern United States J Gen Virol 1996 77 1271 1276 10.1099/0022-1317-77-6-1271 8683216

[B9] Ropp SL Mahlum Wees CE Fang Y Nelson EA Rossow KD Bien M Arndt B Preszler S Steen P Christopher-Hennings J Collins JE Benfield DA Faaberg KS Characterization of emerging European-like porcine reproductive and respiratory syndrome virus isolates in the United States J Virol 2004 78 3684 3703 10.1128/JVI.78.7.3684-3703.2004 15016889 PMC371078

[B10] Chen N Cao Z Yu X Deng X Zhao T Wang L Liu Q Li X Tian K Emergence of novel European genotype porcine reproductive and respiratory syndrome virus in mainland China J Gen Virol 2011 92 880 892 10.1099/vir.0.027995-0 21216986

[B11] Thanawongnuwech R Amonsin A Tatsanakit A Damrongwatanapokin S Genetics and geographical variation of porcine reproductive and respiratory syndrome virus (PRRSV) in Thailand Vet Microbiol 2004 101 9 21 10.1016/j.vetmic.2004.03.005 15201029

[B12] Nam E Park CK Kim SH Joo YS Yeo SG Lee C Complete genomic characterization of a European type 1 porcine reproductive and respiratory syndrome virus isolate in Korea Arch Virol 2009 154 629 638 10.1007/s00705-009-0347-3 19296201

[B13] BioEdit [http://www.mbio.ncsu.edu/BioEdit/bioedit.html]

[B14] Madson DM Patterson AR Ramamoorthy S Pal N Meng XJ Opriessnig T Reproductive failure experimentally induced in sows via artificial insemination with semen spiked with porcine circovirus type 2 Vet Pathol 2009 46 707 716 10.1354/vp.08-VP-0234-O-FL 19276045

[B15] Ullrey DE Sprague JI Becker DE Miller ER Growth of the swine fetus J Anim Sci 1965 24 711 717 14313732 10.2527/jas1965.243711x

[B16] Yoon IJ Joo HS Goyal SM Molitor TW A modified serum neutralization test for the detection of antibody to porcine reproductive and respiratory syndrome virus in swine sera J Vet Diagn Invest 1994 6 289 292 7948196 10.1177/104063879400600326

[B17] Kim W-I Lee D-S Johnson W Roof M Cha S-H Yoon K-J Effect of genotypic and biotypic differences among PRRS viruses on the serologic assessment of pigs for virus infection Vet Microbiol 2007 123 1 14 10.1016/j.vetmic.2007.03.007 17467931

[B18] Zuckermann FA Garcia EA Luque ID Christopher-Hennings J Doster A Brito M Osorio F Assessment of the efficacy of commercial porcine reproductive and respiratory syndrome virus (PRRSV) vaccines based on measurement of serologic response, frequency of gamma-IFN-producing cells and virological parameters of protection upon challenge Vet Microbiol 2007 123 69 85 10.1016/j.vetmic.2007.02.009 17376612

[B19] Meier WA Galeota J Osorio FA Husmann RJ Schnitzlein WM Zuckermann FA Gradual development of the interferon-gamma response of swine to porcine reproductive and respiratory syndrome virus infection or vaccination Virology 2003 309 18 31 10.1016/S0042-6822(03)00009-6 12726723

[B20] Diaz I Darwich L Pappaterra G Pujols J Mateu E Immune responses of pigs after experimental infection with a European strain of Porcine reproductive and respiratory syndrome virus J Gen Virol 2005 86 1943 1951 10.1099/vir.0.80959-0 15958672

[B21] Han K Seo HW Shin JH Oh Y Kang I Park C Chae C Effect of the modified live porcine reproductive and respiratory syndrome virus (PRRSV) vaccine on European and North American PRRSV shedding in semen from infected boars Clin Vaccine Immunol 2011 18 1600 1607 10.1128/CVI.05213-11 21832096 PMC3187033

[B22] Wasilk A Callahan JD Christopher-Hennings J Gay TA Fang Y Dammen M Reos ME Torremorell M Polson D Mellencamp M Nelson E Nelson WM Detection of U.S., Lelystad, and European-like porcine reproductive and respiratory syndrome viruses and relative quantitation in boar semen and serum samples by real-time PCR J Clin Microbiol 2004 42 4453 4461 10.1128/JCM.42.10.4453-4461.2004 15472293 PMC522289

[B23] Gagnon CA del Castillo JR Music N Fontaine G Harel J Tremblay D Development and use of a multiplex real-time quantitative polymerase chain reaction assay for detection and differentiation of *Porcine circovirus-2* genotypes 2a and 2b in an epidemiological survey J Vet Diagn Invest 2008 20 545 558 10.1177/104063870802000503 18776085

[B24] Cheon DS Chae C Comparison of virus isolation, reverse transcription-polymerase chain reaction, immunohistochemistry, and in situ hybridization for the detection of porcine reproductive and respiratory syndrome virus from naturally aborted fetuses and stillborn piglets J Vet Diagn Invest 2000 12 582 587 10.1177/104063870001200619 11108465

[B25] Oleksiewicz MB Bøtner A Madsen KG Storgaard T Sensitive detection and typing of porcine reproductive and respiratory syndrome virus by RT-PCR amplification of whole viral genes Vet Microbiol 1998 64 7 22 10.1016/S0378-1135(98)00254-5 9874099 PMC7117142

[B26] Han K Seo HW Oh Y Kang I Park C Chae C Comparison of the virulence of European and North American genotypes of porcine reproductive and respiratory syndrome virus in experimentally infected pigs Vet J 2013 195 313 318 10.1016/j.tvjl.2012.06.035 22831992

[B27] Halbur PG Paul PS Frey ML Landgraf J Eernisse K Meng XJ Andrews JJ Lum MA Rathje JA Comparison of the antigen distribution of two US porcine reproductive and respiratory syndrome virus isolates with that of the Lelystad virus Vet Pathol 1996 33 159 170 10.1177/030098589603300205 8801709

[B28] Mengeling WL Lager KM Vorwald AC Safety and efficacy of vaccination of pregnant gilts against porcine reproductive and respiratory syndrome Am J Vet Res 1999 60 796 801 10407469

[B29] Benson JE Yaeger MJ Lager KM Effect of porcine reproductive and respiratory syndrome virus (PRRSV) exposure dose on fetal infection in vaccinated and nonvaccinated swine Swine Health Prod 2000 8 155 160

[B30] Labarque GG Nauwynck HJ van Woensel PAM Visser N Pensaert MB Efficacy of an American and a European serotype PRRSV vaccine after challenge with American and European wild-type strains of the virus Vet Res 2000 31 97 10.1051/vetres:2000026

[B31] van Woensel PAM Liefkens K Demaret S Effect on viraemia of an American and a European serotype PRRSV vaccine after challenge with European wild-type strains of the virus Vet Rec 1998 142 510 512 10.1136/vr.142.19.510 9618874

[B32] Roca M Gimeno M Bruguera S Segalés J Díaz I Galindo-Cardiel IJ Martínez E Darwich L Fang Y Maldonado J March R Mateu E Effect of challenge with a virulent genotype II strain of porcine reproductive and respiratoiry syndrome virus on piglets vaccinated ith an attenuated genotype I strain vaccine Vet J 2012 193 92 96 10.1016/j.tvjl.2011.11.019 22264642

[B33] Díaz I Gimeno M Darwich L Navarro N Kuzemtseva L López S Galindo I Segalés J Martín M Pujols J Mateu E Characterization of homologous and heterologous adaptive immune responses in porcine reproductive and respiratory syndrome virus infection Vet Res 2012 43 30 10.1186/1297-9716-43-30 22515169 PMC3403850

[B34] Martínez-Lobo FJ Diez-Fuertes F Simarro I Castro JM Prieto C Porcine Reproductive and Respiratory Syndrome Virus isolates differ in their susceptibility to neutralization Vaccine 2011 29 6928 6940 21807060 10.1016/j.vaccine.2011.07.076

[B35] Osorio FA Galeota JA Nelson E Brodersen B Doster A Wills R Zuckermann F Laegreid WW Passive transfer of virus-specific antibodies confers protection against reproductive failure induced by a virulent strain of porcine reproductive and respiratory syndrome virus and establishes sterilizing immunity Virology 2002 302 9 20 10.1006/viro.2002.1612 12429512

[B36] Lopez OJ Oliveira MF Alvarez Garcia E Kwon BJ Doster A Osorio FA Protection against porine reproductive and respiratory syndrome virus (PRRSV) infection through passive transfer of PRRSV-neutralizing antibodies is dose dependent Clin Vaccine Immunol 2007 14 269 275 10.1128/CVI.00304-06 17215336 PMC1828847

[B37] Scortti M Prieto C Simarro I Castro JM Reproductive performance of gilts following vaccination and subsequent heterologous challenge with European strains of porcine reproductive and respiratory syndrome virus Theriogenology 2006 66 1884 1893 10.1016/j.theriogenology.2006.04.043 16806451

[B38] Lager KM Mengeling WL Brockmeier SL Evaluation of protective immunity in gilts inoculated with the NADC-8 isolate of porcine reproductive and respiratory syndrome virus (PRRSV) and challenge-exposed with an antigenically distinct PRRSV isolate Am J Vet Res 1999 60 1022 1027 10451216

[B39] Bautista EM Molitor TW IFN gamma inhibits porcine reproductive and respiratory syndrome virus replication in macrophages Arch Virol 1999 144 1191 1200 10.1007/s007050050578 10446652

[B40] Rowland RR Robinson B Stefanick J Kim TS Guanghua L Lawson SR Benfield DA Inhibition of porcine reproductive and respiratory syndrome virus by interferon-gamma and recovery of virus replication with 2-aminopurine Arch Virol 2001 146 539 555 10.1007/s007050170161 11338389 PMC7087212

[B41] Diaz I Darwich L Pappaterra G Pujols J Mateu E Different European-type vaccines against porcine reproductive and respiratory syndrome virus have different immunological properties and confer different protection to pigs Virology 2006 351 249 259 10.1016/j.virol.2006.03.046 16712895

[B42] Ferrari L Martelli P Saleri R De Angelis E Cavalli V Bresaola M Benetti M Borghetti P Lymphocyte activation as cytokine gene expression and secretion is related to the porcine reproductive and respiratory syndrome virus (PRRSV) isolate after *in vitro* homologous and heterologous recall of peripheral blood mononuclear cells (PBMC) from pigs caccinated and exposed to natural infection Vet Immunol Immunopathol 2013 151 193 206 10.1016/j.vetimm.2012.11.006 23228653

[B43] Lowe JE Husmann R Firkins LD Zuckermann FA Goldberg TL Correlation of cell-mediated immunity against porcine reproductive and respiratory syndrome virus with protection against reproductive failure in sows during outbreak of porcine reproductive and respiratory syndrome in commercial herds J Am Vet Med Assoc 2005 226 1707 1711 10.2460/javma.2005.226.1707 15906573

[B44] Boehringer Ingelheim[http://www.bi-vetmedica.com]

